# Microvesicles from *Lactobacillus reuteri* (DSM-17938) completely reproduce modulation of gut motility by bacteria in mice

**DOI:** 10.1371/journal.pone.0225481

**Published:** 2020-01-07

**Authors:** Christine L. West, Andrew M. Stanisz, Yu-Kang Mao, Kevin Champagne-Jorgensen, John Bienenstock, Wolfgang A. Kunze

**Affiliations:** 1 McMaster Brain-Body Institute, St. Joseph’s Healthcare, Hamilton, ON, Canada; 2 Department of Biology, McMaster University, Hamilton, ON, Canada; 3 Department of Pathology and Molecular Medicine, McMaster University, Hamilton, ON, Canada; 4 Department of Medicine, McMaster University, Hamilton, ON, Canada; 5 Department of Psychiatry and Behavioural Neurosciences, McMaster University, Hamilton, ON, Canada; Washington State University - Spokane, UNITED STATES

## Abstract

Microvesicles are small lipid, bilayer structures (20–400 nm in diameter) secreted by bacteria, fungi, archaea and parasites involved in inter-bacterial communication and host-pathogen interactions. *Lactobacillus reuteri* DSM-17938 (DSM) has been shown to have clinical efficacy in the treatment of infantile colic, diarrhea and constipation. We have shown previously that luminal administration to the mouse gut promotes reduction of jejunal motility but increases that in the colon. The production of microvesicles by DSM has been characterized, but the effect of these microvesicles on gastrointestinal motility has yet to be evaluated. To investigate a potential mechanism for the effects of DSM on the intestine, the bacteria and its products have here been tested for changes in velocity, frequency, and amplitude of contractions in intact segments of jejunum and colon excised from mice. The effect of the parent bacteria (DSM) was compared to the conditioned media in which it was grown, and the microvesicles it produced. The media used to culture the bacteria (broth) was tested as a negative control and the conditioned medium was tested after the microvesicles had been removed. DSM, conditioned medium, and the microvesicles all produced comparable effects in both the jejunum and the colon. The treatments individually decreased the velocity and frequency of propagating contractile cluster contractions in the jejunum and increased them in the colon to a similar degree. The broth control had little effect in both tissues. Removal of the microvesicles from the conditioned medium almost completely eradicated their effect on motility in both tissues. These results show that the microvesicles from DSM alone can completely reproduce the effects of the whole bacteria on gut motility. Furthermore, they suggest a new approach to the formulation of orally active bacterial therapeutics and offer a novel way to begin to identify the active bacterial components.

## Introduction

Eukaryotic and prokaryotic microorganisms including bacteria, archaea, fungi, and parasites all produce and release spherical membrane vesicles referred to throughout the literature by such names as outer membrane vesicles (OMV), exosomes or microvesicles, depending on their origin [1]. Bacterial membrane vesicles, referred to in this paper as microvesicles (μV), are small spherical structures (20–400 nm) that bleb from the membrane of both Gram-negative and Gram-positive bacteria [2]. They may contain macromolecules as varied as phospholipids, proteins, lipopolysaccharides, peptidoglycans, carbohydrates and nucleic acids [3,4]. Microvesicles may play roles in inter-bacterial communication, bacterial survival, biofilm formation, response to environmental cues, virulence factors, toxins and pathogenic invasion of the host [1,5]. Most of the early work in this area was done on Gram negative bacteria, particularly pathogenic bacteria, such as *Escherichia coli* [6], that release OMVs containing virulence factors to the host [5]. Shen et al. in 2012 showed that the OMV from *B*. *fragilis*, a pathobiont, could by themselves recapitulate *in vivo* the immune downregulatory effects of the parent bacteria [7]. Some of the first evidence that Gram-positive bacteria produce microvesicles was characterized in a study by Lee et al. (2009) using *Staphylococcus aureus*. The bacteria produced μV into the extracellular fluid similar in density and size to those of Gram-negative bacteria with a total of 90 identified protein components [8]. Since then, several studies have characterized the production of membrane vesicles by Gram-positive bacteria, particularly from *Lactobacillus* species [9–12] _._ Similarly, we have demonstrated previously that the μV from a beneficial Gram-positive bacteria, *L*, *rhamnosus* JB-1 (JB-1), reproduced the immune and neuronal functional activities of JB-1, *in vivo*, *ex vivo* and *in vitro* [9]. These studies convincingly show that bacterial μV may have important properties in terms of gut bacteria-host communication and are at least one pathway whereby bacteria may regulate host physiology.

*Lactobacillus reuteri* DSM-17938 (DSM) is a commercially available probiotic and daughter strain of *L*. *reuteri* ATCC 55730, originally isolated from breast milk [13]. This original strain was found to carry potential antibiotic resistance traits and so these plasmids were removed, resulting in DSM [14]. In clinical trials, ingestion of DSM has shown promising results in the management and reduction of gastrointestinal (GI) stress related to diarrhea, constipation, and colic [15–19]. Infants who ingested DSM experienced a significant reduction in the amount of crying and fussing related to colic in comparison to infants receiving a placebo [15,16]. In hospitalized children suffering from acute gastroenteritis, ingestion of DSM reduced the duration of acute diarrhea after 24 h [17]. Administration of DSM increased bowel frequency in infants with functional chronic constipation over several weeks [18]. DSM has also been shown to reduce gastric distention, accelerate gastric emptying, and reduce the frequency of regurgitation in infants with functional gastro-esophageal reflux [19]. We have shown elsewhere that luminal perfusion of mouse jejunal and colonic segments *ex vivo* decreased jejunal contractile motility and increased that of the colon [20]. However, the mechanism whereby DSM modulates these functions has yet to be determined, so the present study was initiated to establish the possible role of μV in this form of interkingdom communication. We isolated the μV from conditioned media in which DSM bacteria were grown, in order to compare their effect on GI motility to that of the parent bacteria. We also compared these activities to those of the conditioned media in which bacteria had been removed and the effects of the medium alone. In these experiments we have shown clearly that bacterial μV may represent a major pathway through which DSM exerts its effects on gut motility.

## Materials and methods

### Animals

Adult male Swiss Webster mice (6–8 weeks) were obtained from Charles River Laboratories (Wilmington, MA, USA). Animals were housed 4-5/cage on a 12-hour light/dark cycle and provided food and water *ad libitum*. The subsequent procedures took place *in vitro*, following cervical dislocation, approved by and in accordance with the McMaster Animal Ethics Research Board (AREB) (permit 16-08-30) [20].

### Tissue flotation bath recordings

Tissue flotation bath recordings were performed as described previously [20]. A minimum of four-centimeter long jejunum and colon segments were extracted and mounted within a tissue flotation bath filled with oxygenated Krebs at 34°C. The oral end of the segments was cannulated, and the contents were flushed from the lumen by gravity perfusion with Krebs. Once clear, the anal end of the segments was cannulated to the silicon outflow tube. The intraluminal compartment was perfused with room temperature Krebs at 5 mL/min. The serosal compartment was perfused by 34°C heated oxygenated Krebs at a rate of 2 mL/min. Oxygenated Krebs was composed of (mmol L^-1^): 118 NaCl, 4.8 KCl, 25 NaHCO_3_, 1.0 NaH_2_PO_4_, 1.2 MgSO_4_, 11.1 glucose, and 2.5 CaCl_2_ bubbled with carbogen gas (95% O_2_ and 5% CO_2_). Prior to recording, the intraluminal pressure was adjusted to 2–3 hPa by increasing and decreasing the heights of the inflow and outflow tubes. Treatments were applied by opening and closing the respective stopcocks to stop intraluminal flow of Krebs and begin flow of bacteria.

### Video recording

Videos were recorded using a JVC video webcam placed 7 cm above the tissue segment. The video clips were recorded and saved in a MOV file format at a frame rate of 10 fps and an aspect ratio of 4:3 using NCH Debut Video Capture. Recording duration varied from 20 minutes to 30 minutes. Using VideoPad Video Editor, the videos were zoomed to four centimeters using a forced aspect ratio of 4:3. The video was converted to black and white by adjusting the colour curves and applying a two-tone filter. The black and white video was exported at 10 fps at a resolution of 400 x 300 pixels.

### Analysis

All generation, manipulation, and analysis of spatiotemporal diameter maps were performed as described in Wu et al. (2013). The video recordings were analyzed using an StMap plugin for NIH Image J software. Using an edge detection routine, the diameter of each position across the gut was represented as a hue value from 0 to 255. Contractions of the gut where the diameter is smaller, approach a hue value of 0, and are represented as darker black areas. Areas of dilation or relaxation approach a hue value of 255 and are white. The software generates a spatiotemporal map throughout the duration of the video. The map displays alternating dark and light hues based on position along the gut, time, and diameter. The spatiotemporal map runs oral to anal on the vertical axis and across time on the horizontal axis. Propagating contractile complexes (PCC) velocity was determined by measuring the slope of the large dark contractions. PCC frequencies were determined by measuring the number of contractions between intervals. Amplitude was measured as the height (gut diameter) of peak contractions. Before and after treatment statistical comparisons were made using paired, two-tailed t-tests using Graphpad Prism software (Version 7.0).

### Bacteria and treatment preparations

DSM from stock was cultured or donated by BioGaia (Stockholm, Sweden) and its μV isolated as described in Al-Nedawi et al (2015). Bacteria were diluted in Krebs to a working concentration of 10^8^ x CFU/mL. Bacteria used for isolation of μV were cultured for 72-hr reaching final concentration of 10^12^ x CFU/mL. Bacteria were subsequently centrifuged at 1900 g (3000 rpm) (IEC Centra GP8R) for 30 min at 4°C. The supernatant was filtered through a 0.2 μm filter. Some supernatant media (CM) was saved for future analysis. The remaining culture medium was centrifuged at 42,000 rpm (Beckman Coulter Type 45 Ti rotor) for 6-hr at 4°C and the supernatant collected and subsequently tested as the conditioned medium minus the μV (CM- μV). Remaining media was cultured and determined to be free of bacteria since no growth occurred over 48 hours. The pellet was collected in phosphate-buffered saline (PBS) washed 3 times and centrifuged for 3-hr at 42,000 rpm (Beckman Coulter Type 70.1 Ti rotor). The remaining μV (pellet) were re-suspended in PBS at a concentration roughly equivalent to 10^12^ CFU/mL and stored at -80°C until use. Microvesicles were diluted to a working concentration roughly equivalent to that generated by bacteria at a final concentration of 10^8^ CFU/mL.

### Nanoparticle tracking analysis

Nanoparticle tracking analysis (NTA) was performed with a NanoSight LM14 (Malvern Panalytical, UK) to characterize the size distribution and concentration of μV suspensions. Samples were diluted in phosphate-buffered saline to a concentration where 30–100 particles per frame were visible, then were flowed through the NanoSight (532 nm laser) using a continuous flow syringe pump. Five 60-second videos were captured (camera level 16) and analyzed using NTA software (v. 3.1, build 3.1.54; Malvern Panalytical) with a detection threshold of 5.

## Results

**Fig 1:**
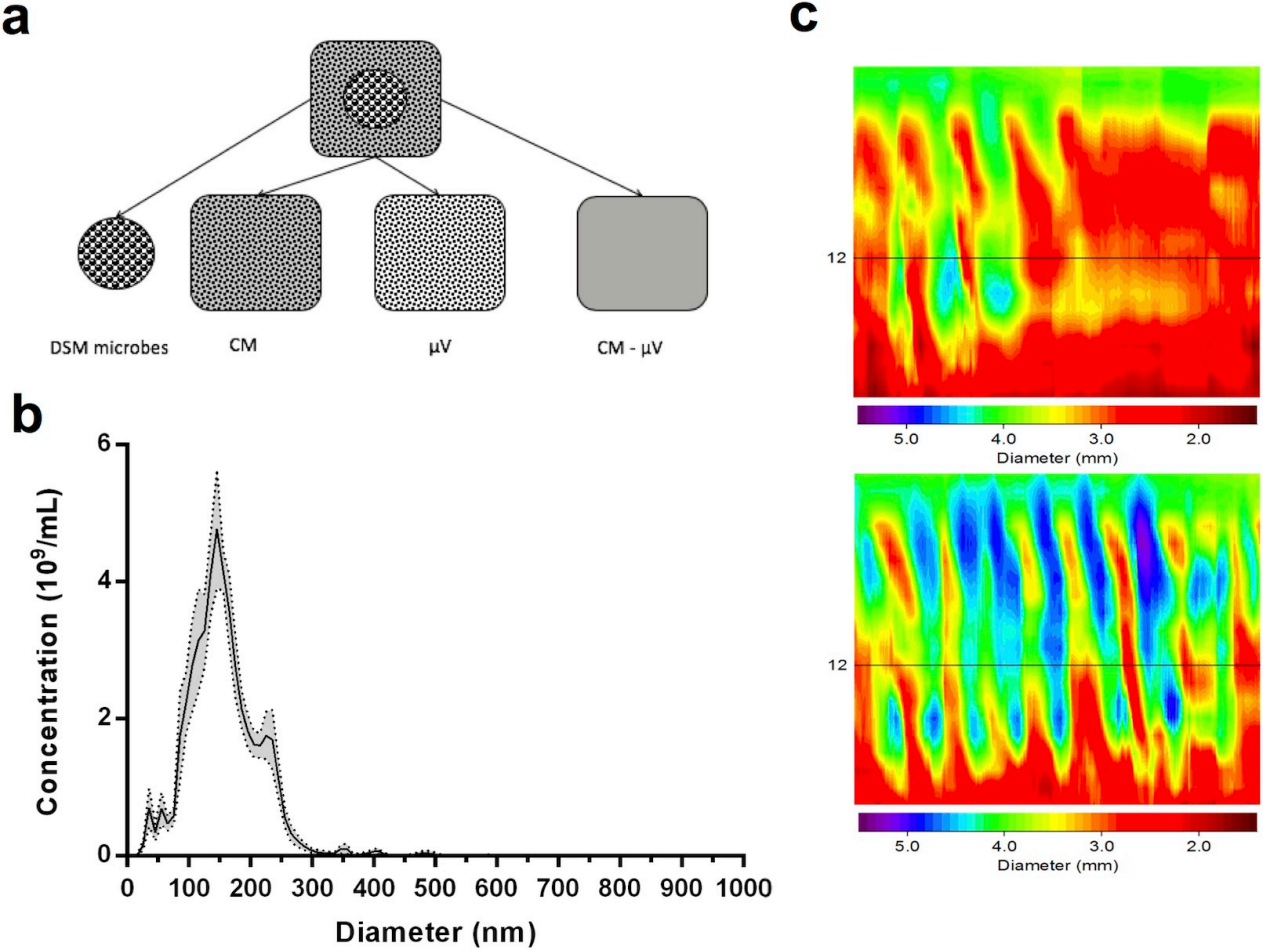
The experimental design describing the protocol for DSM bacteria and μV isolation. **a)** The *Lactobacillus reuteri* DSM-17938 whole bacteria (large black spheres) were removed from the growth medium and washed. The remaining conditioned medium (grey square including small black dots representing μV), with bacteria removed, was applied separately. Microvesicles (small black dots) were removed from the conditioned medium and applied separately. The remaining conditioned medium (empty grey square) after removal of μV was applied separately. **b)** μV size distribution and concentration for individual preparations were estimated by nanoparticle tracking analysis. In the representative example shown here, the average particle size was 156.3 ± 2.1 nm and the modal particle size was 139.3 +/- 7.6 nm. The total estimated concentration of nanoparticles was 5x10^10^ per mL. Data are binned in 10 nm increments. Error ribbon (grey shadow) depicts standard error of the mean. **c)** Spatiotemporal heat map example of propagating contractile clusters (PCC) in the colon during Krebs control (top) and after intraluminal treatment with DSM-17938 (bottom). Contractions of the colon (smaller diameters) are represented by red-yellow and relaxations (larger diameters) are represented by green-blue. Contractions are propagated from oral to anal ends of the tissue across the y-axis and time is represented by the x-axis. Intraluminal DSM increased the velocity and frequency of colonic propagating contractions.

DSM and the products of its cultivation were applied intraluminally to *in vitro* preparations of mouse jejunum and colon to determine whether μV could replicate the effect of the parent bacteria on intestinal motility (Fig 1A). The μV were characterized via NTA as described in the methods. The isolated microvesicles had an average size of 156.3 ± 2.1 nm and the modal size was 139.3 +/- 7.6 nm (+/- SEM) (Fig 1B). The remaining conditioned media after the μV and bacteria had been removed (CM-μV) was then administered intraluminally to the tissue. As a negative control, the growth media used to culture the bacteria (broth) was applied separately. The effect of these treatments was compared to Krebs buffer control and measured across three parameters of propagating contractile complexes (PCC) in the gut segments: velocity, frequency, and amplitude (Fig 1C).

### *L*. *reuteri* (DSM) and its products decreased small intestinal motility

#### Jejunal PCC velocity

**Fig 2:**
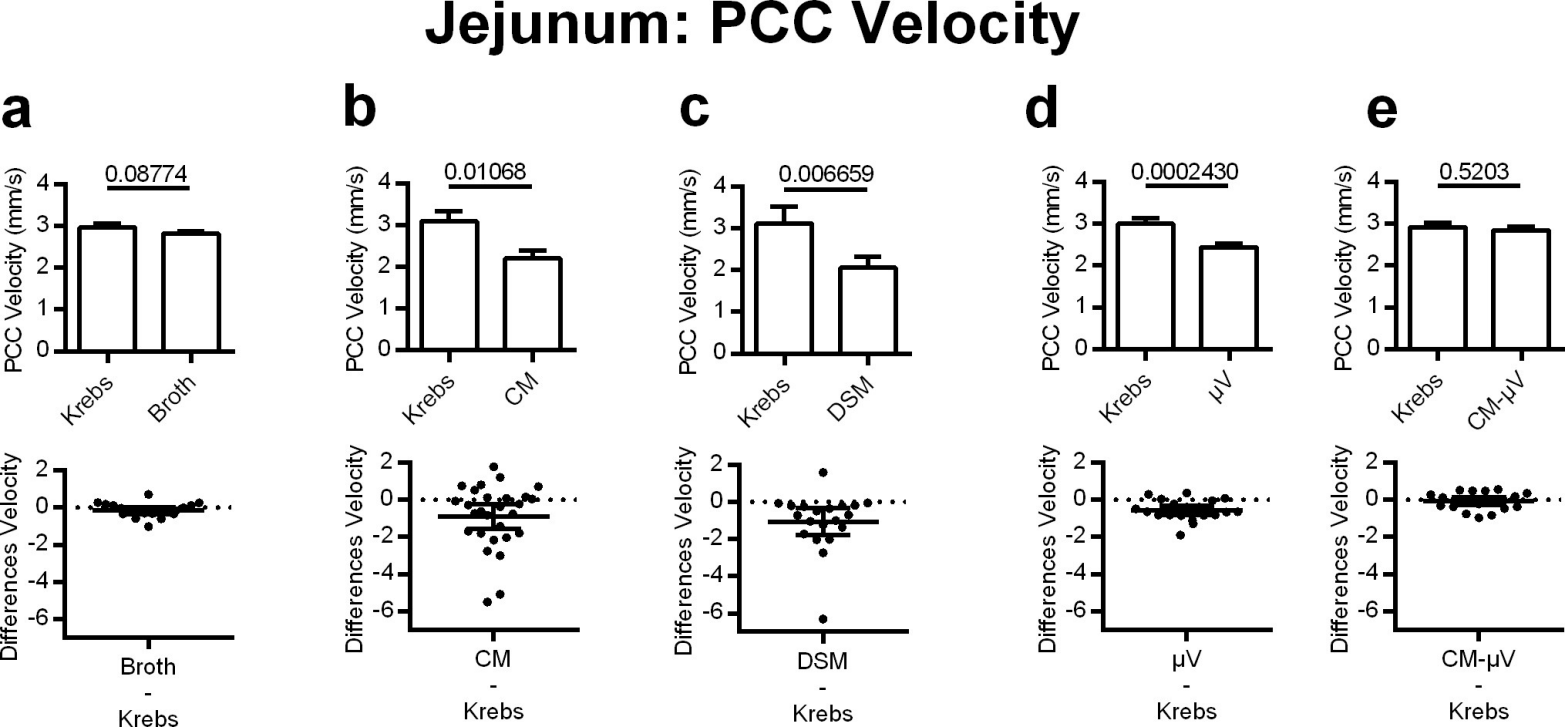
Effects of adding DSM, DSM derived microvesicles μV (μV) or media on PCC velocity for mouse jejunal segments, *in vitro*. Upper panels: bar graphs showing means and standard errors. **c)** DSM (N = 20), **b)** conditioned medium (CM) (N = 28) and **d)** DSM μV reduced PCC velocity (N = 20). Neither **(a)** broth (N = 20) nor conditioned medium minus μV **(e)** (CM-μV) (N = 20) altered PCC velocity. P values derived from paired t-tests are given above horizontal bars. Lower panels: individual value plots of difference (treatment-control Krebs) with 95% confidence intervals for each matching graph in upper row. All subsequent figures show the same relationships between upper and lower panels. Significance determined when p < 0.05.

DSM, CM, and μV all reduced PCC velocity to a similar degree in the jejunum. As a negative control, the broth used as media to culture the bacteria was tested independently and had negligible effect on jejunal PCC velocity (5% decrease) compared to Krebs control (p = 0.0877, n = 20) (Fig 2A). DSM significantly reduced jejunal PCC velocity by 34% when applied intraluminally (p = 0.0067, n = 20) (Fig 2C). The CM recapitulated the effect of the parent bacteria and decreased PCC velocity by 29% (p = 0.0107, n = 28) (Fig 2B). Microvesicles isolated from the 72hr culture significantly decreased jejunal PCC velocity by 19% (p = 0.0002, n = 20) (Fig 2D). The conditioned media after the μV had been removed (CM-μV) did not change jejunal PCC velocity when applied to the lumen (p = 0.5203, n = 20) (Fig 2E).

#### Jejunal PCC frequency

**Fig 3:**
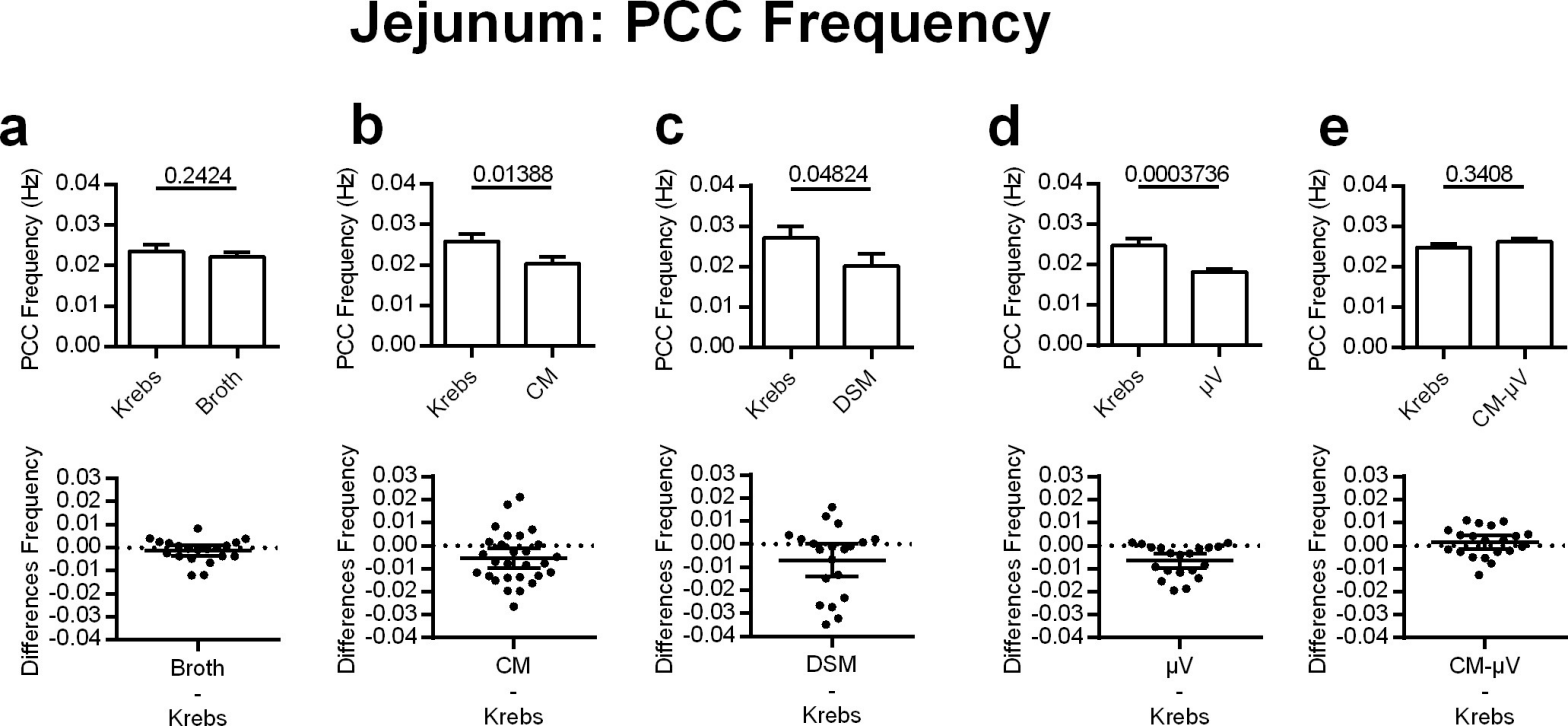
Effects of adding DSM, μV or media on PCC frequency for mouse jejunal segments, *in vitro*. **c)** DSM (N = 20), **b)** CM (N = 28) and **d)** μV (N = 20) decreased PCC frequency. **a)** Broth (N = 20) or **e)** CM-μV (N = 20) had no statistically significant effects on PCC frequency. P values derived from paired t-tests are given above horizontal bars. Significance determined when p < 0.05.

Decreases in PCC frequency in the jejunum were also produced by DSM, CM, and μV. The broth did not significantly change jejunal PCC frequency (6% decrease) when applied to the lumen (p = 0.2424, n = 20) (Fig 3A). DSM significantly reduced PCC frequency by 26% in the jejunum (p = 0.0482, n = 20) (Fig 3C). Similarly, CM decreased PCC frequency by 21% (p = 0.0139, n = 28) (Fig 3B). Microvesicles significantly decreased jejunal PCC frequency by 26% as comparable to the bacteria (p = 0.0004, n = 20) (Fig 3D). Jejunal PCC frequency was not significantly affected by the luminal addition of conditioned media after the μV had been removed (CM-μV) (p = 0.3408, n = 20) (Fig 3E).

#### Jejunal PCC amplitude

**Fig 4:**
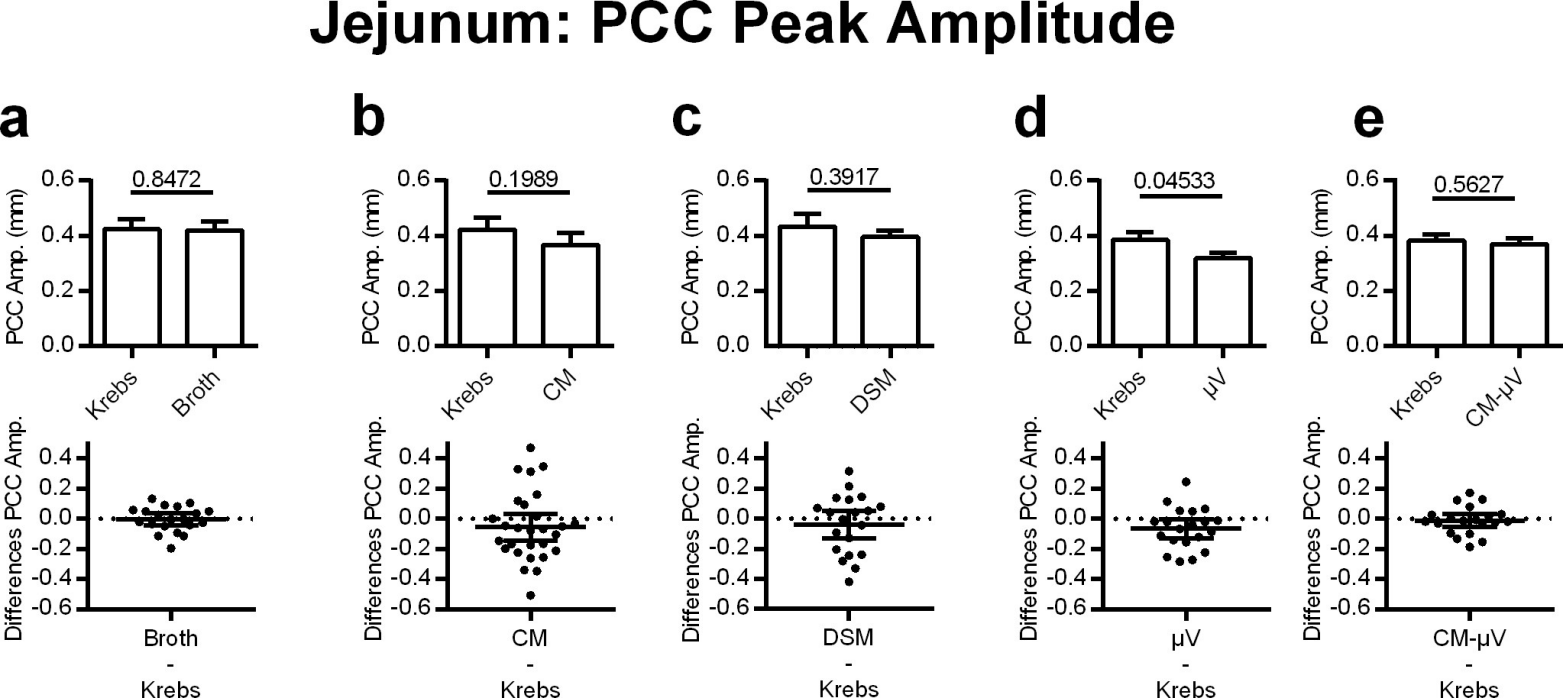
Effects of adding DSM, μV or media on PCC peak amplitude for mouse jejunal segments, *in vitro*. **d)** μV (N = 20) but not **a)** broth (N = 20), **b)** CM (N = 28), **c)** DSM (N = 20), or **e)** CM-μV (N = 20) reduced PCC amplitude. P values derived from paired t-tests are given above horizontal bars. Significance determined when p < 0.05.

PCC amplitude in the jejunum was not significantly altered in any of the treatment groups, with the exception of the μV (Fig 4). Microvesicles decreased jejunal PCC amplitude by 17% (p = 0.0453, n = 20) (Fig 4D), despite this effect not being present in the DSM or CM trials (p = 0.3917, n = 20 and p = 0.1989, n = 28, respectively). Broth and conditioned media after the μV had been removed (CM-μV) did not change jejunal PCC amplitude (p = 0.8472 and p = 0.5627, n = 20) (Fig 4A & 4E).

### *L*. *reuteri* (DSM) and its products increased colonic motility colonic PCC velocity

**Fig 5:**
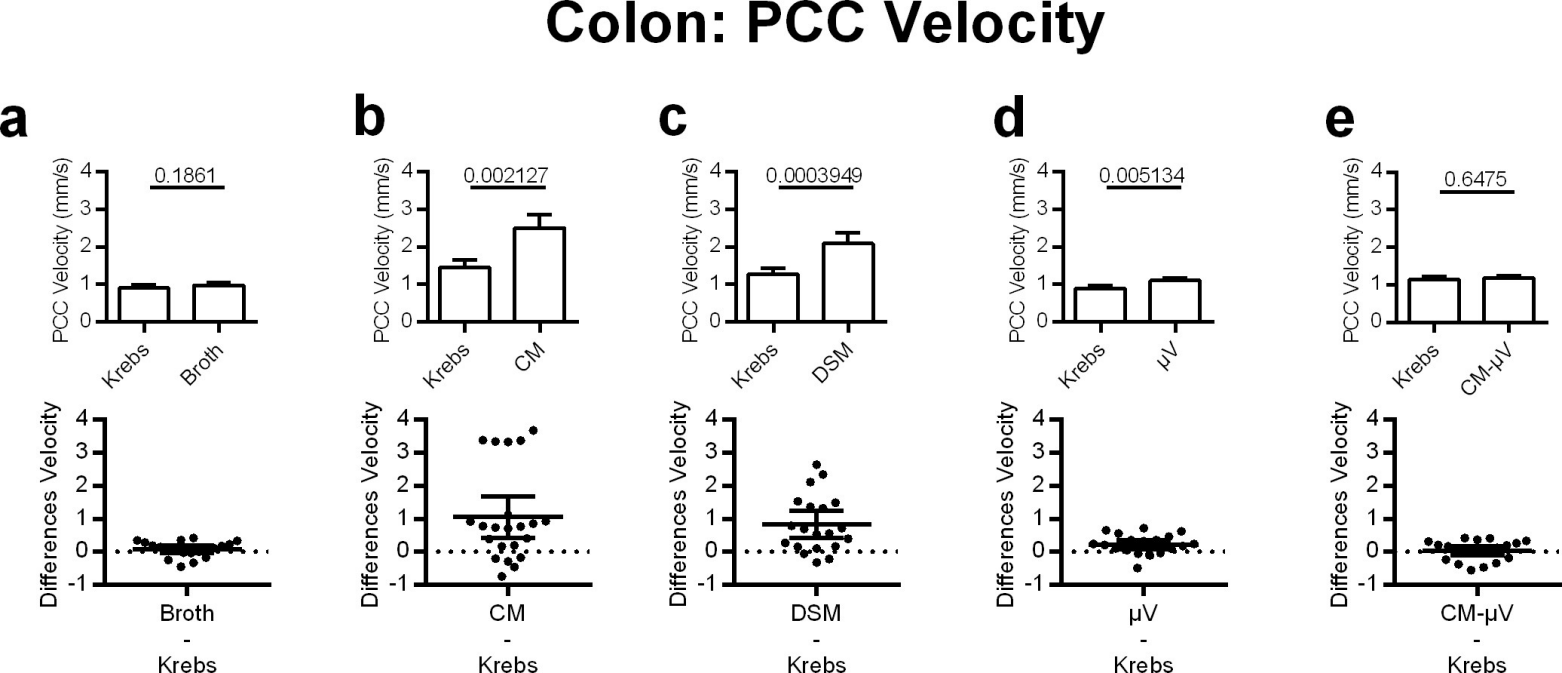
Effects of adding DSM, DSM derived microvesicles or media on PCC velocity for mouse colon segments, *in vitro*. **c)** DSM (N = 20), **b)** CM (N = 28) and **d)** μV (N = 20) increased PCC velocity. **a)** Broth (N = 20) or **e)** CM-μV (N = 20) had no statistically significant effects on PCC velocity. P values derived from paired t-tests are given above horizontal bars. Significance determined when p < 0.05.

Colonic contractile motility was stimulated by the addition of either DSM, CM, or μV. Broth continued to have little effect on intestinal motility, increasing colonic PCC velocity by 8%, but not within the 0.05 significance range (p = 0.1861, n. = 20) (Fig 5A). DSM significantly increased the velocity of PCC contractions in the colon by 65% (p = 0.0004, n = 20) (Fig 5C). This was recapitulated by the CM, which significantly increased PCC velocity by 72% in the colon (p = 0.0021, n = 28) (Fig 5B). Microvesicles significantly increased the velocity of PCCs in the colon by 24% (p = 0.0051, n = 20), but to a lesser degree than that produced by DSM and CM (Fig 5D). Conditioned media after the μV had been removed (CM-μV) applied intraluminally failed to change colonic PCC velocity significantly (p = 0.6475, n = 20) (Fig 5E).

#### Colonic PCC frequency

**Fig 6:**
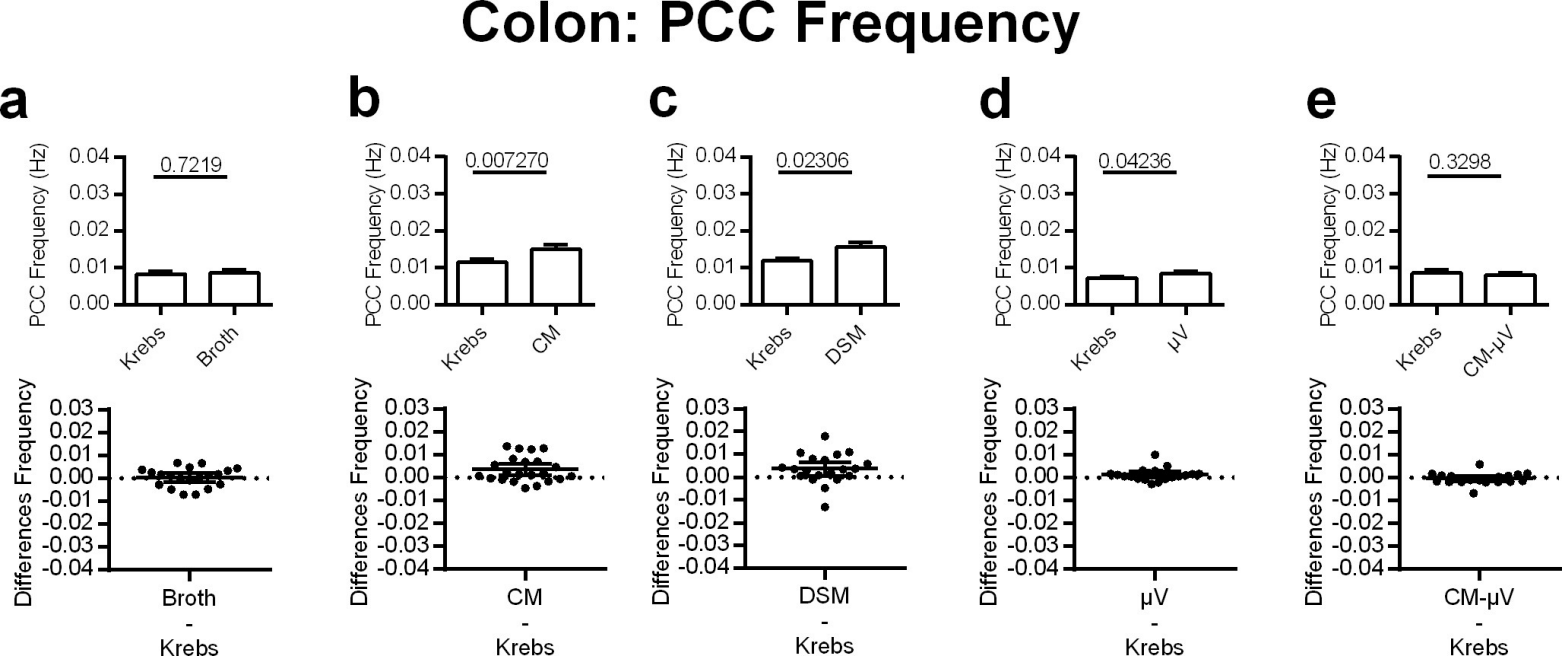
Effects of adding DSM, μV or media on PCC frequency for mouse colon segments, *in vitro*. **c)** DSM (N = 20), **b)** CM (N = 28) and **d)** μV (N = 20) increased PCC frequency. **a)** broth (N = 20) or **e)** CM-μV (N = 20) had no statistically significant effects on PCC frequency. P values derived from paired t-tests are given above horizontal bars. Significance determined when p < 0.05.

DSM, CM, and μV all stimulated colonic motility by increasing the frequency of PCC contractions. The broth increased colonic PCC frequency by a small and insignificant amount (4%), (p = 0.7219, n. = 20) (Fig 6A). DSM significantly increased colonic PCC frequency by 30% (p = 0.0231, n = 20) (Fig 6C). In the same capacity, CM significantly increased PCC frequency by 31% in the colon (p = 0.0073, n = 28) (Fig 6B). Similar to what was seen with PCC velocity, μV increased the frequency of colonic PCCs by 18% (p = 0.0424, n = 20); (Fig 6D). Conditioned media after the μV had been removed (CM-μV) did not significantly affect PCC frequency in the colon (p = 0.3298, n = 20) (Fig 6E).

#### Colonic PCC amplitude

**Fig 7:**
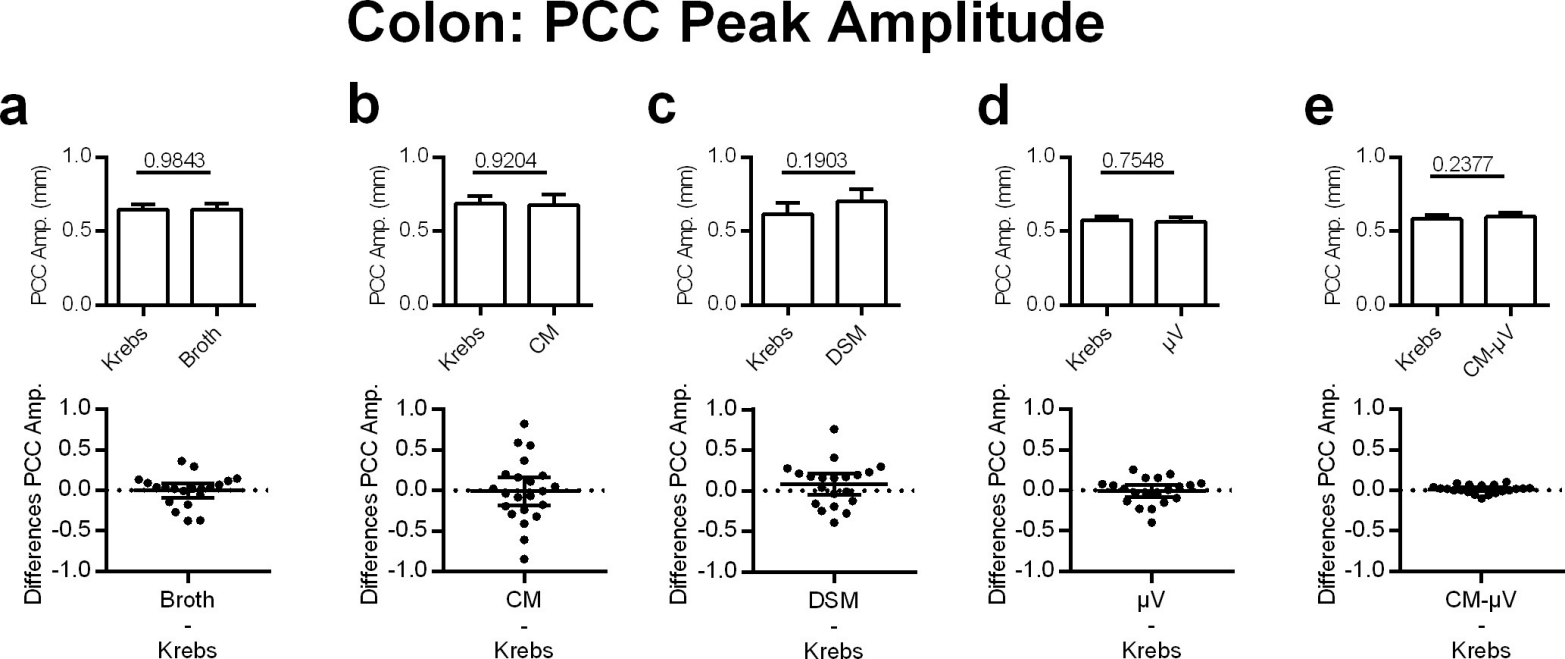
Effects of adding DSM, μV or media on PCC peak amplitude for mouse colon segments, *in vitro*. Neither **a)** broth (N = 20), **b)** CM (N = 28), c**)** DSM (N = 20), **d)** μV (N = 20) nor **e)** CM-μV (N = 20) had statistically significant effects on PCC peak amplitude. P values derived from paired t-tests are given above horizontal bars. Significance determined when p < 0.05.

PCC amplitude in the colon was not significantly affected by DSM or any of the other treatment modalities (Fig 7).

## Discussion

DSM had regional-specific effects on intestinal motility; decreasing jejunal and increasing colonic PCC velocity and frequency of contractions, as previously reported [20]. The present study demonstrates that both the μV and the conditioned media recapitulate the effect of DSM on intestinal motility in both the small intestine and the colon. Furthermore, positive results were not seen when the conditioned media was applied following the removal of the μV (CM-μV), suggesting that the majority of the functional effects are contained in μV. Thus, the μV themselves may be the principal pathway of communication between these gut bacteria and the host. One limitation of the present study, however, was that it was performed on jejunal and colonic samples from healthy adult male mice. Further studies should be performed in females, infant and aged mouse models, and mouse models of pathological dysmotility.

Several publications in addition to those of Shen et al [7] referred to earlier, have shown that conditioned media from bacterial and yeast cultures, similar to that of the CM used in the present study, produce effects in experimental models in the absence of the parent bacteria. Culture medium from *L*. *rhamnosus* GG (LGG) upregulated serotonin transporter (SERT) mRNA and protein levels in the intestine [21], enhanced resistance to *E*. *coli* K1 infection in neonates [22], and improved acute alcohol-induced liver injury and intestinal permeability [23]. Culture supernatants from *L*. *acidophilus* and LGG produced an anti-inflammatory response, inhibiting gene expression of the matrix metalloproteinase (MMP) MMP-9, expression of which has been noted in inflammatory disease [24]. Conditioned media from *L*. *plantarum* inhibited NF-κB binding activity and proteasome function [25]. We have recently shown that culture supernatants from *Saccharomyces cerevisiae* and *boulardii* yeasts reverse acute stress-induced dysmotility in the gut and reproduce the effects seen with the yeasts themselves [26]. Although the presence of μV in these studies was not tested, these results support the suggestion that bioactive molecules or components produced or secreted by yeasts and bacteria are capable of therapeutic effects independent of the parents.

As interest in the various roles of μV grows, research into their capability to produce effects on the GI tract, has emerged. μV produced by *L*. *casei* BL23 contain proteins that have been previously identified as mediators of the effect of *Lactobacillus* probiotics; p40, p75, and LCABL_31160 [27]. Particularly p40 and p75 produced by *L*. *casei* BL23 bind to mucin, collagen, and cultured epithelial cells [28]. We previously showed that JB-1 μV reproduced the same immune and neuronal effects caused by the whole bacteria [9]. Proteomic analysis of JB-1 and its μV showed that GroEL an analogue of heat shock protein, could independently reproduce immune activities of the bacteria [9]. Introduction of JB-1 into the gut lumen decreased the amplitude of neuronally dependent colonic migrating motor complexes subsequently referred to as PCC and this was reproduced by the μVs alone [9]. Microvesicles from *L*. *plantarum* WCFS1 increased survival of *C*. *elegans* infected with *E*. *faecium* (vancomycin resistant *enterococci*) [10]. These results support the role of μV in gut *Lactobacillus* bacterial signaling and their significance in the mechanisms of communication with mammalian hosts within the microbiome-gut-brain axis.

Extensive gut innervation by the enteric nervous system (ENS) suggests it is likely that some of the effects exerted by probiotics target the ENS [29]. Additionally, the PCC evaluated in the present study are considered to be ENS-dependent as they are abolished, as we have shown, by the application of the specific neurotoxin, tetrodotoxin [20,30]. We have previously reported [9] that the activation of the ENS by JB-1 and its μV in the gut lumen, was the result of an unknown additional signaling mechanism to the underlying intrinsic primary afferent neurons (IPAN), invoked in the epithelium. Direct placement of bacteria or μV onto the IPAN failed to elicit action potentials, whereas they did occur if they were independently applied to the epithelium [9]. Bacteria, including probiotics, produce a variety of active substances such as neuromodulators and neurotransmitters that could act directly or indirectly via epithelial cells on neurites in the underlying neuronal cell layer [31,32]. One such study showed that ingestion of *L*. *reuteri* reduced the size of myenteric plexus cell slow after-hyperpolarization (AH) by inhibiting calcium-dependent potassium channel (IK_Ca_) opening [33]. It was predicted from these results that the functional effect of an increase in AH cell excitability may increase the inhibitory tone of the ENS on the circular muscle, decreasing the amplitude of propulsive contractions [33]. The findings of the Wang et al. (2010) study were consistent with this hypothesis [29]. However, we do not know if DSM or its μV also inhibit the IK_Ca_ channel.

The negligible effects of the CM after removal of the μV are compelling. They strongly suggest that a product or membrane component of the bacteria within the μV may produce the therapeutic benefits of the bacteria on gut motility. As discussed previously there are numerous components that have been identified within μV including, outer membrane and periplasmic constituents, proteins, lipoproteins, phospholipids, lipopolysaccharide (LPS), toxins, carbohydrates and signaling proteins [1,3,4], which produce a range of effects. Other recent studies have verified the production of μV from *L*. *reuteri* strains, including the particular strain (DSM) investigated here [11,12]. Grande et al. (2017) identified that μV produced by both planktonic and biofilm phenotypes of the bacteria were associated with eDNA, and their structures prevented its digestion by DNase I [12]. They also showed that proteins and phospholipids are integral to the structure of DSM μVs as these were degraded by Phospholipase C and Proteinase K. However, no evidence was presented for functional effects in host gut.

Given our findings it is likely that commercial formulations of this bacteria and possibly also others that exert their effects in the gut through the microbiome in the microbiota-gut-brain axis, may be advantaged by including μV in their preparation. Our results may also facilitate the identification of specific molecular structures and bacterial components responsible for such beneficial effects.

## Conclusions

The results of this study demonstrate a role for μV in inter-kingdom signaling and substantially decrease the number of possible components that could be responsible for *L*. *reuteri*-related modulation of gut motility. They also point out the advantages of inclusion of μV in bacterial formulations for oral use. The identification of functional activity attributed to the bacteria within or associated with their μV, substantially decreases the number of possible candidate molecules and structural components of potential beneficial bacteria and probiotics that are responsible for their effects. A future study will need to characterize the active components of the μV involved in modulation of gut motility.

## Supporting information

S1 File (XLSX)
